# Links between blood parasites, blood chemistry, and the survival of nestling American crows

**DOI:** 10.1002/ece3.4287

**Published:** 2018-08-07

**Authors:** Andrea K. Townsend, Sarah S. Wheeler, David Freund, Ravinder N. M. Sehgal, Walter M. Boyce

**Affiliations:** ^1^ Department of Biology Hamilton College Clinton New York; ^2^ Sacramento‐Yolo Mosquito and Vector Control District Elk Grove California; ^3^ Department of Biology San Francisco State University San Francisco California; ^4^ Department of Pathology, Microbiology, and Immunology School of Veterinary Medicine University of California Davis California

**Keywords:** acute infection, avian health parameters, avian malaria, ecoimmunology, immunocompetence, pathogenicity

## Abstract

Many studies have used the avian hemosporidians (*Leucocytozoon*,* Plasmodium*, and *Hemoproteus*) to test hypotheses of host–parasite co‐evolution, yet documented health and survival consequences of these blood parasites vary among studies and generalizations about their pathogenicity are debatable. In general, the negative effects of the hemosporidians are likely to be greatest during acute infections of young birds, yet most previous studies in wild passerines have examined chronic effects in adults. Here, we evaluated responses of nestling American crows (*Corvus brachyrhynchos*) to acute infection (prevalence and burden), as well as its short‐ and long‐term survival consequences. We used panel of nine hematological and biochemical parameters that are regularly used to evaluate the health of domestic animals, including leukocyte profiles, hematocrit, and plasma proteins. We assessed the effects of infection on survival in a mark‐recapture framework. Overall, 56% of crows (*n *=* *321 samples) were infected by at least one of the three genera. Infections by all genera were associated with elevated plasma proteins and globulins, which could indicate an adaptive immune response. However, only *Plasmodium* infections were associated with low hematocrit (anemia) and lower fledging success, possibly mediated by the negative effect of low hematocrit values on body condition. Moreover, early *Plasmodium* infection (<40 days of age) had long‐term survival implications: it was associated with lower apparent survival probability within 3 years after fledging. These results suggest that young crows mounted an adaptive immune response to all three genera. Short‐ and long‐term pathological effects, however, were only apparent with *Plasmodium* infections.

## 1 INTRODUCTION

Disease‐causing parasites have the potential to place selection pressure on natural host populations and to play a major role in host ecology and evolution (Anderson & May, 1982; Poulin, 2007). Theoretical and empirical data indicate that parasites may promote the evolution and maintenance of genetic diversity (Anderson & May, 1982), favor the evolution of sexually selected traits (Hamilton & Zuk, 1982), and drive host population cycles (Hudson, Dobson, & Newborn, 1998). The avian hemosporidian parasites in the genera *Leucocytozoon*,* Plasmodium*, and *Hemoproteus* have been used as a model system in the study of such host–parasite interactions (Atkinson & Van Riper, 1991; Hamilton & Zuk, 1982; Poulin, Marshall, & Spencer, 2000; Zuk & Borrello, 2013), yet their documented costs—and, potentially, the magnitude of selection pressure that they exert—appear to vary widely among hosts, populations, and contexts. They can have devastating impacts in naïve populations (Atkinson & Samuel, 2010), and many studies have reported negative associations with infection in endemic areas, including reductions in condition (Marzal, Bensch, Reviriego, Balbontin, & de Lope, 2008; Merino, Moreno, Sanz, & Arriero, 2000), antipredator behavior (Garcia‐Longoria, Moller, Balbontin, de Lope, & Marzal, 2015; Mukhin et al., 2016), mating display behavior (Bosholn, Fecchio, Silveira, Braga, & Anciaes, 2016), survival (Asghar et al., 2015; Krams et al., 2013; Sol, Jovani, & Torres, 2003), and reproductive output (Asghar, Hasselquist, & Bensch, 2011; Knowles, Palinauskas, & Sheldon, 2010; Marzal et al., 2013; Merino et al., 2000). Other studies, however, have reported no associations—or even positive associations—with infection (Cornelius, Davis, & Altizer, 2014; Fargallo & Merino, 2004; Piersma & van der Velde, 2012; Podmokla et al., 2014; Zylberberg et al., 2015), or have reported effects that vary among host species (Atkinson & Van Riper, 1991; Ellis, Kunkel, & Ricklefs, 2014; Sorci, 2013), host population (Piersma & van der Velde, 2012), parasite species (Asghar et al., 2011; Lachish, Knowles, Alves, Wood, & Sheldon, 2011; Marzal et al., 2008), and characteristics of individual hosts (Hammers et al., 2016). As such, the extent to which the hemosporidians broadly constitute a selective pressure across different systems and contexts is unclear.

The stage of infection at which the hemosporidian parasites are evaluated will affect the apparent severity of their costs. In general, the pathological effects of infection are expected to be higher at the initial stage of infection (acute infection) than at chronic stages (described in Atkinson & Van Riper, 1991; LaPointe, Atkinson, & Samuel, 2012; Valkiūnas, 2005). In the acute phase, parasites increase in number until they reach peak levels (referred to as the crisis stage), at which point the physiological stresses of infection are likely to be the greatest. Birds that survive the acute phase then enter the chronic phase, during which parasites persist at low levels (potentially for the lifetime of the bird). Endemic hematozoa are therefore likely to exert their highest costs during acute infections (Atkinson & Van Riper, 1991; Sol et al., 2003; Williams, 2005), yet most studies in wild passerines focus on chronic infections in adults rather than acute infections in nestlings. Costs (or lack thereof) measured during the chronic stage of infection could therefore underestimate the strength of selection that the hematozoa place on their hosts.

**Figure 1:**
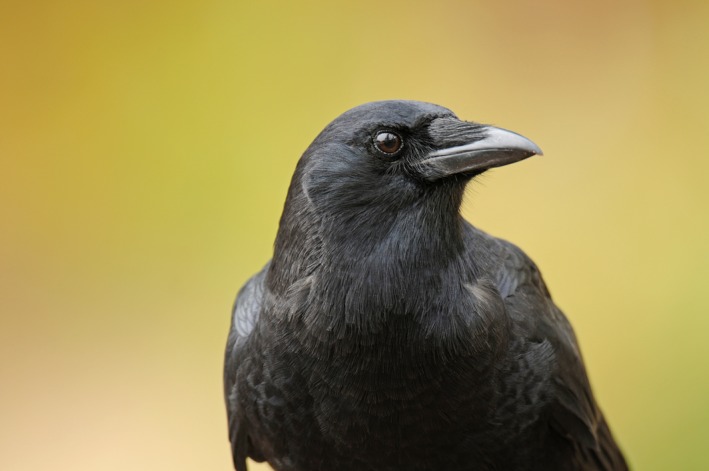
The American crow (*Corvus brachyrhynchos*) [Colour figure can be viewed at http://wileyonlinelibrary.com]

In this study, we examined the effects of acute hemosporidian parasite (*Leucocytozoon*,* Plasmodium*, and *Hemoproteus*) infection on American crow (*Corvus brachyrhynchos*; Figure 1) nestlings. Specifically, we evaluated the links between hemosporidian infection and a suite of health indices in nestling crows (Table 1). These indices included nine hematological and biochemical parameters that are regularly used to evaluate the health of domestic animals (Campbell & Ellis, 2007), but which have seldom been used in studies of acute hemosporidian infections of wild passerines (Krams et al., 2013). These parameters include white blood cell counts and profiles (Ellis et al., 2014; Norte, Araujo, Sampaio, Sousa, & Ramos, 2009; Schoenle, Kernbach, Haussmann, Bonier, & Moore, 2017); heterophil:lymphocyte (H:L) ratios (Krams et al., 2013; Norte et al., 2009), hematocrit (LaPointe et al., 2012; Sorci, 2013; Williams, 2005), plasma proteins (albumin, globulin), and their ratio (Alb:Glo) (Kilgas, Tilgar, & Mand, 2006; Ots, Murumagi, & Horak, 1998; Williams, 2005). We also examined links between infection and (1) a body condition index, which is a common nonspecific index of health used in ecological studies, and which has been linked to survival probability and signs of other pathogenic infections in crows (Townsend, Clark, McGowan, Miller, & Buckles, 2010), (2) fledging success, to evaluate short‐term links between infection and survival, and (3) apparent survival up to 3 years postfledging, to evaluate longer‐term links between early infection and survival. Brief descriptions of the health parameters that we measured and their predicted relationships with parasitic infections are given in Table 1.

**Table 1:** Parameters, their descriptions, and their predicted relationships with parasitic infections

Parameter	Description	Prediction (parasitized)	Source
Total WBC count	Basis of immune system; elevated during infection	Higher	Krams et al. (2013), Norte et al. (2009), Ots et al. (1998)
Lymphocytes	Assist in recognition and destruction of specific pathogens	Higher	Ellis et al. (2014, Krams et al. (2013, Ots et al. (1998)
Heterophils	Phagocytic cells; proliferate during infections; can damage host tissues	Higher	Campbell & Ellis (2007), Davis et al. (2008), Ellis et al. (2014), Krams et al. (2013), Ots et al. (1998)
H:L ratio	Ratio of heterophils to lymphocytes; indicator of stress & inflammatory response	Higher	Krams et al. (2013), Norte et al. (2009)
Hematocrit	Percentage of red blood cells in whole blood; rupture of infected erythrocytes can lower hematocrit and induce anemia	Lower	Atkinson & Van Riper (1991), LaPointe et al. (2012), Norte et al. (2009), Schoenle et al. (2017), Sorci (2013), Williams(2005)
Plasma protein	Transport and immune function; decreases associated with disease	Lower	Ots et al. (1998)
Albumin	Plasma protein; decreases associated with pathological states and malnutrition	Lower	Kilgas et al. (2006), Ots et al. (1998)
Globulins	Plasma protein; includes immunoglobulins that bind antigens	Higher	Atkinson & Van Riper (1991), Ots et al. (1998), Williams(2005)
Alb:Glo	Lower ratio indicate disease states	Lower	Ots et al. (1998)
Body condition	Weight by size residual	Lower	Marzal et al. (2008), Williams(2005)
Fledging success	Successful departure from nest	Lower	Krams et al. (2013)
Post‐fledging survival	Apparent survival after accounting for detection probability	Lower	Asghar et al. (2015), Sol et al. (2003)

Notes

H:L* *= heterophil:lymphocyte ratio; Alb:Glo* *=* *albumin:globulin ratio.

## 2 MATERIALS AND METHODS

### 2.1 Study system: American crows and the hemosporidian parasites

American crows (“crows” hereafter) are long‐lived corvids that breed in cooperative groups generally comprising a socially monogamous pair and 0–10 auxiliary birds (Townsend, Clark, McGowan, & Lovette, 2009). Natal philopatry is high in this species, and auxiliaries are usually adult offspring from previous broods. Hemosporidian parasites have complex life cycles, described in Valkiūnas (2005). In brief, all three taxa are transmitted to their avian hosts by hematophagous Diptera [*Hemoproteus* typically by biting midges (Ceratopogonidae) and hippoboscid flies (Hippoboscidae); *Plasmodium* typically by mosquitoes (Culicidae); *Leucocytozoon* typically by blackflies (Simuliidae)]. *Plasmodium* undergoes merogony (asexual reproduction) in the erythrocytes, a process that ruptures the host red blood cells. In contrast, merogony occurs in other tissues (e.g., heart, liver, kidney) in the life cycle of *Leucocytozoon* and *Hemoproteus*. In all three genera, the gametocytes develop in blood cells (erythrocytes for *Plasmodium* and *Hemoproteus*, and both red and white blood cells for *Leucocytozoon*), which are then ingested by their respective insect vectors during bloodfeeding.

### 2.2 Field sampling and monitoring

We sampled crows on the urban campus of the University of California, Davis (38°53′90.60″N, 121°75′72.94″W), and into the surrounding campus‐owned agricultural areas (e.g., vineyards, pastures, and row crops (Townsend & Barker, 2014)). Blood samples were collected from 240 crows from 104 nests in Davis, CA, from 2012 to 2014 (*n *=* *67, 105, and 68 nestlings from 32, 43, and 29 nests in 2012, 2013, and 2014, respectively). These 104 nests were produced by 74 unique crow family groups. Birds were sampled 0–40 days after hatching, either as nestlings within their nests or as young fledglings on branches immediately adjacent to the nests. Nests were situated on lateral tree branches and accessed by boom lift. Surviving nestlings that were sampled <18 days after hatching were sampled a second time >22 days after hatching because they were too small to band at the time of initial sampling. Crows <18 days old were individually marked with a unique toenail clip; crows >18 days old were marked with both a numbered USGS band and a unique color band. We also measured body mass, tarsus length, bill width, bill depth, and head size to estimate body condition. Age (±~3 days) was estimated from approximate hatch date (inferred from the shifting and probing behavior of incubating females, as well as size and feather development of nestlings) following criteria used in Townsend et al. (2010).

Blood samples were taken from the jugular vein using 27 gauge ½‐inch needles and 1‐mL syringes. One drop was used to make a blood smear, which was air‐dried, fixed with methanol for one minute, and stained with Giemsa (SIGMA Diagnostics #48900). Another drop was placed in lysis buffer until extraction. The remaining blood (~300 μl) was stored on ice in heparinized tubes for hematological and biochemical analyses, carried out within 5 hr at the Veterinary Medical Teaching Hospital Clinical Diagnostic Laboratories at the University of California, Davis.

After sampling and banding, we monitored marked birds for fledging (successful departure from the nest) and postfledging survival 1–7 times per week along established census routes (Hinton, Reisen, Wheeler, & Townsend, 2015; Taff & Townsend, 2017; Townsend & Barker, 2014; Wheeler et al., 2014). All field work was performed under protocols approved by the Institutional Animal Care and Use Committee of the University of California, Davis (protocol #16897).

### 2.3 Parasite screening: microscopy

We screened for *Leucocytozoon*,* Plasmodium*, and *Hemoproteus* prevalence (defined as the presence or absence of infection) and burden (defined as the proportion of infected cells) using 1,000× light microscopy (oil immersion). *Plasmodium* and *Hemoproteus* burden was estimated by counting the number of parasites in 1,000 red blood cells, whereas *Leucocytozoon* was estimated as the number per 1,000 red and white blood cells. Slides were also evaluated at low power (200×–400×) for five minutes to screen for low‐intensity infections. Burden was estimated as percentage of infected cells per 1,000 red (or red and white for *Leucocytozoon*) blood cells. Samples with very low burdens, for which parasites were only detected during the five‐minute scans, were scored as having a burden of 0.05%. Slides were screened by two technicians who worked simultaneously and cross‐checked scores for consistency. Birds were classified here as having “single infections” when infected by only one genus; they were classified as “co‐infected” when infected by more than one genus.

### 2.4 Parasite screening: molecular

Molecular scoring of *Leucocytozoon*,* Plasmodium*, and *Hemoproteus* followed methods described in Freund et al. (2016). In brief, genomic DNA was extracted from whole blood (DNeasy Blood and Tissue extraction kits; Qiagen, Valencia, CA) and screened for the three hemosporidian parasites using a nested PCR described in Hellgren, Waldenstr, & Bensch (2004), using modified PCR conditions (Freund et al., 2016). Each PCR reaction was run in 25‐μl volumes, accompanied by a positive control that was previously verified by sequencing and microscopy and a negative control of purified water. The PCR amplicons were visualized on a 1.8% agarose gel using ethidium bromide staining, and samples with parasite cyt *b* amplification were purified with ExoSAP (United States Biochemical Corporation, Cleveland, OH). We identified lineages by sequencing the fragments bidirectionally using the BigDye version 1.1 sequencing kit (Applied Biosystems, Inc., Foster City, CA) on an ABI PRISM3100^™^ automated sequencer (Applied Biosystems). Sequences were edited in Sequencher 4.9 (GeneCodes, Ann Arbor, MI), aligned by eye in MacClade 4.08a (Maddison and Maddison 2005), and then subjected to a BLAST search to ensure that the expected genus was amplified.

### 2.5 Hematologic and biochemical testing

Analyses were performed within five hours of blood collection at the Veterinary Medical Teaching Hospital Clinical Diagnostic Laboratories, University of California, Davis, following Vergneau‐Grosset, Polley, Holt, Vernau, & Paul‐Murphy (2016). In brief, hematocrit was determined by centrifugation of blood at 10,000 g in a Sorvall Legend Micro 17 Microcentrifuge (Thermo Scientific Corp, Waltham, MA, USA). White blood cells counts were determined manually by certified clinical laboratory scientists using Natt and Herrick solution with 0.5% new methylene blue stain and a Neubauer ruled hemocytometer. Counts were crosschecked by manual estimates on blood smears, averaging leukocyte counts from 10 microscopic fields (×50) and multiplying the mean by 2,500 to determine a leukocyte estimate per microliter. Differential 200‐cell leukocyte counts were performed manually on Wright‐stained smears stained with an automatic stainer (Wescor Inc., Logan, UT, USA). Total plasma protein was determined by a manual refractometer reading by a laboratory technician. Plasma biochemical panels were evaluated using a Roche Cobas c501 analyzer (Roche Diagnostics, Indianapolis, IN, USA).

### 2.6 Statistical analyses

#### 2.6.1 Links between infection and blood parameters

*Prevalence*. To examine the relationship between parasite prevalence and the different hematological parameters, we used linear mixed‐effects models fit by REML, using package NLME in R v3.3.1 (R Core Team, 2016). The H:L ratios were positively skewed and were log‐transformed to meet assumptions of normality. All other parameters approximated a normal distribution. We ran a series of eight models, specifying WBC count, lymphocytes (%), heterophils (%), log(H:L ratio), hematocrit, plasma protein, globulin, albumin, or their ratios (Alb:Glo) as the response variables. We specified prevalence (0/1) of *Leucocytozoon*,* Plasmodium*, and *Hemoproteus* as fixed effects and family group as a random effect to account for nonindependence among offspring produced by the same family. We included year, age, and sex as fixed effects, but sequentially removed them from the models when they were not significant following the model‐selection criteria of Hosmer & Lemeshow (2000). To determine if results were consistent when birds were co‐infected (infected by more than one genus) as opposed to exhibiting single infections, we re‐ran the prevalence models with infection status (none, single, or mixed) instead of the three unique genera.

*Burden*. To examine the relationship between parasitemia and hematological parameters, we ran the same set of eight models, specifying burden (% infected cells) of each genus instead of prevalence. As before, we specified family as a random effect and sex, year, and age as fixed effects, sequentially removing them from the models when they were not significant.

#### 2.6.2 Links between infection, blood parameters, condition, and short‐term survival

We assessed direct, short‐term links between infection and (a) body condition index and (b) short‐term survival probability (fledging success). Body condition was estimated as the residual from a quadratic regression of weight regressed against (body size)^2^. Body size was defined as the first principal component on covariances of four morphological measurements (tarsus, head, and bill width and depth); this component explained 98% of the total variation in these measurements. We considered individuals with positive residuals to be in better condition (i.e., heavier than expected given their skeletal size). This index of body condition has been correlated with disease‐mediated mortality and inbreeding in a previous study of American crows (Townsend et al., 2010).

We examined direct effects of parasites on body condition index in a linear mixed effects model (as above), with either the prevalence or burden of each parasite as predictors, while specifying family as a random effect and sex, age, and year as fixed effects. As above, we re‐ran the prevalence model with infection status (none, single, or mixed) to assess the effects of infection by more than one genus.

Fledging success was defined as successful departure of crows from their nests. We examined fledging success (0/1; binomial distribution, logit link) as a function of infection in a generalized linear mixed model (library MASS in R), specifying either prevalence or burden of each genus (or co‐infection) as fixed effects, family as a random effect and sex, age, and year as fixed effects.

To evaluate indirect links with infection, we ran two additional post hoc tests. First, we examined body condition as a function of the blood parameters that were consistently linked to different infections. We then examined fledging success as a function of these parameters and body condition, which was itself indirectly linked to infection (see Section 3). Model frameworks used in these analyses were the same as those described above.

#### 2.6.3 Links between early infection and post‐fledging survival

We used mark–recapture analyses to evaluate longer‐term associations between early acute infection and survival after fledging. Analyses were restricted to the birds that successfully fledged their nests. Capture‐history matrices for these birds were based on resight data, collected 1–7 days per week from May of 2012 until July of 2015. Birds were followed for 14–38 months after hatching, depending on the year in which they hatched. Capture histories were collapsed into 13 encounter intervals. Multiple resights within intervals were treated as a single encounter.

Apparent survival (φ) and recapture (*p*) parameters were estimated in the program MARK v. 8.0 (http://www.phidot.org/software/mark/index.html). Following Lebreton, Burnham, Clobert, & Anderson (1992), model selection was made using the Akaike information criterion (AIC; Akaike, 1974). Preliminary tests indicated that an underlying model with a fully time‐dependent φ and time‐constant p was the best‐supported model for birds after fledging. Model fit was verified by dividing the deviance estimate by the mean of the simulated deviances from a parametric goodness‐of‐fit test (1000 bootstrap samples). Specifying this model [φ(*t*)*p*(.)] as the underlying model, we generated models to detect infection prevalence [*Leucocytozoon* (*L*), *Plasmodium* (*P*), and *Hemoproteus* (*H*)] effects on φ, starting with [φ(t+*L*+*P*+*H*)p(.)] as the global model. The model set was balanced with respect to the factors of primary interest [φ (*L*), φ (*P*), and φ (*H*)]. Models with lower AIC scores, corrected for finite sample size (AIC_c_), were considered as the more parsimonious models given the data.

## 3 RESULTS

### 3.1 General results and descriptive statistics

In total, 321 samples were examined for *Leucocytozoon*,* Plasmodium*, and *Hemoproteus*. These samples were collected from 240 individual crows from 74 family groups. Eighty‐one birds were sampled twice because they were too small to band at the time of initial sampling, with a minimum of three days between sampling periods (mean ± *SE *=* *13.58 ± 0.61 days between sampling periods; range 3–25 days). Hematology and blood chemistry data were obtained for a subset of these samples, with sample size differing slightly among measurements (range: 145–175 measurements for each of the different parameters). Mean values of these parameters are given in the supplementary materials (Supporting information Table S1).

Infections were scored by PCR [*n *=* *262/321 (81.6%) samples] for prevalence, or microscopy [*n *=* *185/321 (57.6%) samples] for both prevalence and burden; a subset of 126 samples were scored by both methods. When results were discordant (e.g., when an infection was detected by PCR that was not visible by microscopy; 15.8% of scores), we gave precedence to the molecular score because this assay has a lower threshold of detection (Valkiūnas et al., 2008); we scored the burden for these samples as very low (0.05%). Burden was scored as zero for samples that tested negative by PCR and for which microscopy had not been conducted. We could not score all samples by both methods because we did not collect both slides and blood samples at every sampling point. It is possible that some cases of infection were undetectable (e.g., in latent infections, or when parasitemia levels were extremely low; Valkiūnas et al. 2008), particularly among individuals scored only by microscopy.

**Figure 2:**
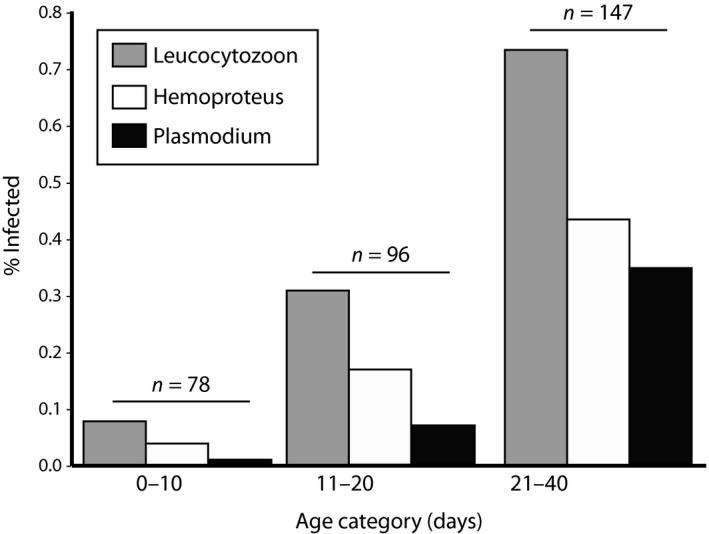
Prevalence of hemosporidian parasites among the different age categories (1–10, 11–20, and 21–40 days after hatching) of crows. Sample sizes indicated above each age category. Percentages with age class are shown here for the purpose of illustration. Model details are given in the text

Overall, 56% of samples were infected by at least one of the three genera and 31% were infected by more than one genus. Percentages of samples that tested positive for *Leucocytozoon*,* Plasmodium*, and *Hemoproteus* were 44%, 18%, and 26%, respectively. Prevalence increased with age (range: 0–40 days) at time of sampling for all three genera [generalized linear models with occurrence (0/1) as the response and age as the predictor; *p *<* *0.001; *n *=* *321]. Patterns of increasing infection prevalence with age class are illustrated in Figure 2.

In all subsequent analyses, we restricted our dataset to the last available measurements (infection prevalence and burden, with corresponding hematological and condition data at that time point) collected from each individual nestling. We restricted our dataset to the last available measurement per individual because this represented the most complete, inclusive measurement of infection status for each individual in the nestling stage, as infections accumulated (and were not lost) in individuals over the course of the nestling period.

### 3.2 Links between infection and blood parameters

Results of statistical tests comparing infection prevalence and burden with different blood parameters are given in Tables 2, 3. Full model results, showing effects of year, age, and sex (when significant) are given in the supplementary materials (Supporting information Tables S2, S3). In general, we observed either no statistical correlation (e.g., with WBC profiles) or statistically significant results (e.g., hematocrit; plasma proteins) that matched our predictions, summarized in Table 1.

**Table 2:** Effects of infection prevalence on health state parameters of American crow nestlings

Parameter	*n*	*Leucocytozoon*	*p*	*Hemoproteus*	*p*	*Plasmodium*	*p*	Co‐infections
WBC count	126/54	−2384 ± 2213.01	0.29	4489.42 ± 2392.81	0.06	−140.88 ± 2467.87	0.95	ns
Heterophils	126/54	−2.09 ± 2.81	0.46	6.91 ± 3.28	**0.04**	−1.15 ± 3.29	0.73	ns
Lymphocytes	126/54	2.83 ± 3.4	0.41	−3.27 ± 3.98	0.41	1.87 ± 3.99	0.64	ns
H:L	126/54	−0.07 ± 0.09	0.44	0.17 ± 0.11	0.12	−0.06 ± 0.11	0.60	ns
Hematocrit	130/53	0.23 ± 0.81	0.78	−0.53 ± 1	0.60	−2.02 ± 0.99	**0.046**	ns
Plasma protein	135/54	0.27 ± 0.11	**0.02**	0.47 ± 0.12	**>0.001**	0.59 ± 0.13	**>0.001**	***
Albumin	135/54	0.06 ± 0.06	0.30	0.02 ± 0.07	0.78	−0.01 ± 0.07	0.92	ns
Globulin	135/54	0.17 ± 0.1	0.11	0.43 ± 0.11	**>0.001**	0.41 ± 0.12	**>0.001**	***
Alb: Glo	135/54	−0.07 ± 0.05	0.17	−0.17 ± 0.06	**0.003**	−0.12 ± 0.06	0.06	***
Condition	199/68	4.00 ± 5.03	0.43	−3.84 ± 5.59	0.49	1.11 ± 5.95	0.85	ns
Fledging	199/68	0.25 ± 0.4	0.53	−0.08 ± 0.47	0.87	−0.95 ± 0.46	**0.04**	ns

Notes

Sample size (*n*) given as number of individuals/number of nests sampled. Values given for each parasite indicate effect sizes (β ± *SE*) of infection. Full model results, showing effects of year, age, and sex (when significant) are given in the supplementary materials (Supporting information Table S2).

H:L* *= heterophil:lymphocyte ratio; Alb:Glo* *=* *albumin:globulin ratio.

Significant p‐values (α <0.05) indicated in bold font.

**Table 3:** Effects of parasite burden on health state parameters of American crow nestlings

Parameter	*n*	*Leucocytozoon*	*p*	*Hemoproteus*	*p*	*Plasmodium*	*p*
WBC count	115/50	−3878.13 ± 2673.13	0.15	−471.5 ± 276.49	0.09	−354.11 ± 370.09	0.34
Heterophils	115/50	−2.67 ± 3.41	0.44	0.58 ± 0.35	0.11	0.78 ± 0.49	0.12
Lymphocytes	115/50	1.61 ± 3.93	0.68	−0.36 ± 0.41	0.39	−0.27 ± 0.53	0.62
H:L*	115/50	−0.05 ± 0.11	0.64	0.01 ± 0.01	0.29	0.02 ± 0.01	0.26
Hematocrit	122/50	0.92 ± 0.83	0.27	0.02 ± 0.09	0.84	−0.51 ± 0.12	**>0.001**
Plasma protein	127/51	−0.01 ± 0.12	0.90	0.06 ± 0.01	**>0.001**	0.04 ± 0.02	**0.01**
Albumin	128/52	−0.01 ± 0.11	0.96	0.01 ± 0.01	0.29	−0.02 ± 0.01	**0.02**
Globulin	128/52	0.05 ± 0.17	0.79	0.05 ± 0.01	**>0.001**	0.05 ± 0.01	**>0.001**
Alb:Glo*	128/52	−0.002 ± 0.1	0.98	−0.02 ± 0.01	**0.02**	−0.02 ± 0.01	**0.02**
Condition	194/66	−7.56 ± 6.33	0.23	−0.16 ± 0.51	0.75	−1.30 ± 0.79	0.10
Fledging	206/67	−0.23 ± 0.55	0.68	−0.04 ± 0.04	0.39	0.08 ± 0.09	0.36

Notes

Sample size (*n*) given as number of individuals/number of nests sampled. Values given for each parasite indicate effect sizes (β ± *SE*) with burden. Full model results, showing effects of year, age, and sex (when significant) are given in the supplementary materials (Supporting information Tables S3).

H:L* *= heterophil:lymphocyte ratio; Alb:Glo* *=* *albumin:globulin ratio.

Significant p‐values (α <0.05) indicated in bold font.

#### 3.2.1 Prevalence

**Figure 3:**
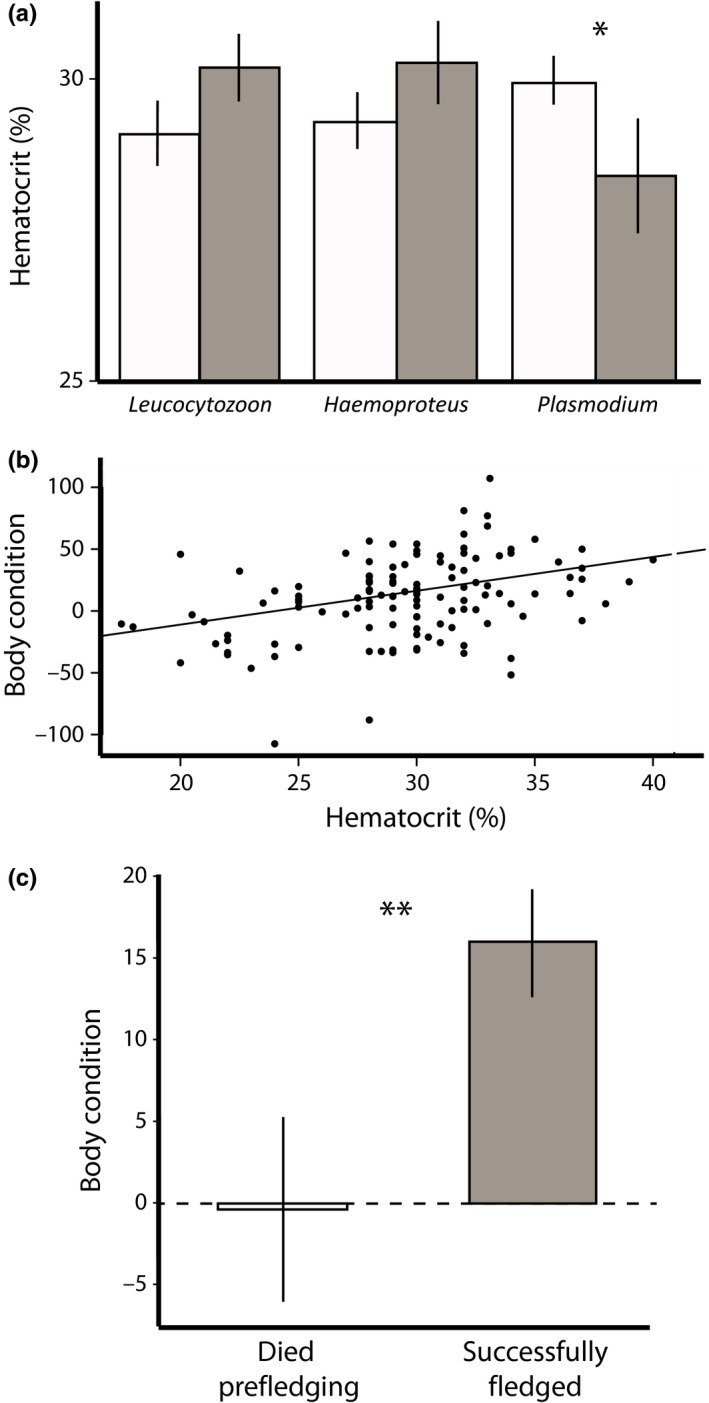
Links between *Plasmodium* prevalence, hematocrit, condition, and survival. (a) Mean hematocrit level among birds with (gray bars) and without (white bars) *Plasmodium* infections (*n *=* *130 birds). (b) Body condition as a function of hematocrit levels (*p *<* *0.001; *r* ^2^ **=* *0.13; *n *=* *123 birds). (C) Mean body condition of birds that fledged successfully (gray bars; *n *=* *77 birds) and birds that did not fledge successfully (white bars; *n *=* *46 birds). Simple bivariate relationships shown here for the purpose of illustration (*α < 0.05; ***α < 0.001). Full model details are given in the text [Colour figure can be viewed at http://wileyonlinelibrary.com]

There was no link between the prevalence of any of the three parasites with WBC counts, lymphocytes, H:L ratios, or albumin (Table 2). There was a consistent, positive association between elevated plasma protein levels and infection by each of the three parasites. These elevated plasma protein levels appeared to be due to globulin levels, which were significantly higher in birds infected with *Hemoproteus* and *Plasmodium*. Albumin : globulin ratios were only significantly lower in birds infected with *Hemoproteus*, although a trend for lower Alb:Glo ratios was apparent in the other genera. Two patterns were genus specific: Heterophils were significantly higher among birds infected with *Hemoproteus*, and hematocrit was significantly lower among birds infected with *Plasmodium* (Figure 3a).

Similar patterns for plasma proteins and globulins were derived when specifying co‐infection status (none, single, or mixed) instead of specific infections (Table 2; last column). Plasma proteins and globulin levels were significantly higher among infected birds than uninfected birds (*p *<* *0.001). Post hoc tests indicated that birds with co‐infections had higher plasma protein and globulin levels than birds with single infections and uninfected birds; birds with single infections had intermediate levels (Tukey's HSD; α* *= 0.05). The Alb:Glo ratio was higher for infected birds than for uninfected birds (*p *<* *0.001), but post hoc tests indicated no difference between birds infected with a single genus or multiple genera. Co‐infection status was not associated with any of the other hematological parameters (*p *>* *0.05).

#### 3.2.2 Burden

Specifying burden instead of prevalence yielded generally similar results (Table 3). As with prevalence, there was no link between burden of any of the three parasites and WBC counts, lymphocytes, or H:L ratios. Likewise, *Hemoproteus* and *Plasmodium* burden (although not *Leucocytozoon* burden) was associated with elevated plasma protein and globulin levels and higher Alb:Glo ratios. As with prevalence, *Plasmodium* burden was strongly associated with lower hematocrit levels. Differences were few: Heterophils did not vary with burden of any parasite species, and albumin levels were negatively associated with *Plasmodium* burden.

### 3.3 Links between infection, blood parameters, condition, and short‐term survival

*Plasmodium* prevalence (although not burden) was associated with lower fledging success (Tables 2, 3). No other direct links with infection were apparent. Body condition did not vary with prevalence or burden of any parasite, and fledging success was not linked to *Hemoproteus* or *Leucocytozoon* infections.

Impacts of infection could be mediated indirectly through the blood parameters that the parasites affect. The two blood parameters that were most consistently linked to infection were globulin levels (to all genera) and hematocrit (to *Plasmodium*). We therefore examined body condition as a function of globulin and hematocrit (*n *=* *123 birds; 51 nests). Globulin had no effect on body condition (β ± *SE *=* *0.84 ± 4.7; *p *=* *0.86). Hematocrit, however, was positively correlated with body condition (β ± *SE *=* *2.70 ± 0.61; *p *<* *0.001): anemic birds were in relatively poor condition (Figure 3b). We next examined fledging success as a function of globulin, hematocrit, and body condition (*n *=* *123 birds; 51 nests). Condition was significantly, positively associated with fledging success (β ± *SE *=* *0.02 ± 0.01; *p *=* *0.019; Figure 3c). Neither hematocrit (β ± *SE *=* *−0.04 ± 0.06; *p *=* *0.52) nor globulin (β ± *SE *=* *−0.03 ± 0.41; *p *=* *0.95) were predictors of fledging.

### 3.4 Links between nestling infection and postfledging survival

**Figure 4:**
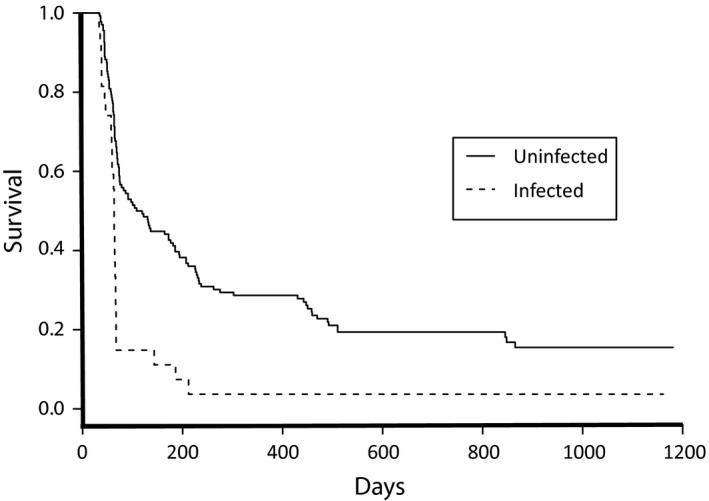
Kaplan–Meier survival plots of American crow fledglings, grouped by nestling *Plasmodium* infection status. Birds that were infected as nestlings (*n *=* *26, dashed line) had lower proportional survival than uninfected birds (*n *=* *111, solid line; log rank: *Χ* ^2^ **=* *18.5; *p *<* *0.001)

In total, 163 of the 240 marked birds (67.9%) successfully fledged. After fledging, apparent survival was lower for birds that had been infected with *Plasmodium* as nestlings, but did not vary with other hemosporidian infections. In mark–recapture analysis, the four models that included *Plasmodium* status (prevalence as nestlings) as a predictor had the most support, given the data (Table 4; cumulative model support: 0.71). In contrast, cumulative model support for an effect of *Hemoproteus* and *Leucocytozoon* infection was relatively weak (0.57 and 0.45, respectively), and inclusion of these terms in the models did not substantially improve model fit (∆AIC_c_ < 2) over a model with *Plasmodium* alone. Kaplan‐Meier curves illustrating the negative relationship between *Plasmodium* infection and postfledging survival are shown in Figure 4.

**Table 4:** Candidate set of approximating models generated to fit postfledging American crow mark–recapture data

Candidate models	AICc	∆AIC_c_	AIC_c_ Weights	np	deviance
φ (*P*+*H*+t) p(.)	477.9533	0	0.20871	13	450.744
φ (*P*+t) p(.)	477.9617	0.0084	0.20783	11	455.0904
φ (*P*+*L+*t) p(.)	478.6087	0.6554	0.15039	13	451.3994
φ (*P*+*H*+*L+*t) p(.)	478.7774	0.8241	0.13823	14	449.3774
φ (*H*+*L*+t) p(.)	479.0396	1.0863	0.12124	12	454.0065
φ (*H*+t) p(.)	479.4694	1.5161	0.0978	12	454.4363
φ (*L+*t) p(.)	481.1106	3.1573	0.04305	11	458.2394
φ (t) p(.)	481.6568	3.7035	0.03276	10	460.9332

Notes

(np: number of parameters; φ: survival; *P*:* Plasmodium* prevalence; *H*:* Hemoproteus* prevalence; *L*:* Leucocytozoon* prevalence; p: recapture; t: time.) Prevalence refers to infection status of nestlings.

## 4 DISCUSSION

The strength of selection pressure that parasites place on their hosts will depend on the extent to which they cause disease in those hosts. Studies that broadly use the avian hematozoa as a model system to address important evolutionary questions about host–pathogen interactions (Atkinson & Van Riper, 1991; Hamilton & Zuk, 1982; Poulin et al., 2000; Zuk & Borrello, 2013) make the underlying assumption that hemosporidian infections cause disease (i.e., that the organisms are pathogenic). Although hemosporidian infections do, indeed, appear costly in some hosts and contexts (Asghar et al., 2015; Atkinson & Van Riper, 1991; Krams et al., 2013; Sol et al., 2003), many studies have failed to detect negative consequences of infection, particularly among chronically infected adults (Cornelius et al., 2014; Fargallo & Merino, 2004; Piersma & van der Velde, 2012; Podmokla et al., 2014; Zylberberg et al., 2015). Here, we examined evidence for pathogenicity during acute infection of young crows, using a panel of hematological and biochemical tests that are often used to evaluate consequences of infection in veterinary and human medicine. We found strong and consistent relationships between several of these blood parameters and acute infection. Specifically, elevated plasma protein and globulin levels were associated with infection (prevalence and/or burden) by all three genera. In addition, infection by *Plasmodium* was associated with low hematocrit levels, a lower probability of fledging from the nest, and a lower postfledging survival probability. Overall, our results suggest that young crows mounted an adaptive immune response to *Leucocytozoon*,* Plasmodium*, and *Hemoproteus* (Ots & Horak, 1998), but that only *Plasmodium* infection was linked to anemia, poor condition, and lower short‐ and long‐term survival probabilities. Among nestling American crows, *Plasmodium* therefore appears to be more pathogenic (and, potentially, may exert stronger selection pressure) than the other genera of hemosporidian parasites.

The current study was unusual in its focus on infection at the nestling stage, when costs of infection are likely to be highest (Atkinson & Van Riper, 1991; Sol et al., 2003; Williams, 2005). Reasons for the more typical focus on adults may be largely logistical, because the time until parasites are detectable in the blood can exceed the nestling period for many passerine species (Hammers et al., 2016; Krams et al., 2013). This study was also unusual in its evaluation of a large panel of hematological and biochemical parameters, which are regularly used to evaluate the health of domestic animals (Campbell & Ellis, 2007), but can be difficult to collect in smaller passerines because of constraints on blood collection imposed by body size. American crows provided a useful system for this study because they are large‐bodied (>300 g) passerines with a relatively long (>30 day) nestling periods; moreover, their high natal philopatry of both sexes (Verbeek & Caffrey, 2002) facilitated the collection of long‐term survival data, which are difficult to collect in most passerines after fledging. In the following sections, we discuss the interpretation of the hematological and biochemical parameters with respect to infection by the avian hematozoa.

### 4.1 Immune responses to infection

The elevated globulin levels that were consistently detected in infected crows can be interpreted as evidence of an adaptive immune response to *Leucocytozoon*,* Plasmodium*, and *Hemoproteus*. The globulin fraction of the plasma protein contains immunoglobulins (Ig), parasite‐specific antibodies that increase after infection (Ots et al., 1998). These results are congruent with a prior study showing that adult great tits (*Parus major*) with chronic *Hemoproteus* infections had higher plasma globulin fractions, which the authors attributed to a humoral (antibody) response to infection (Ots & Horak, 1998). Because these studies are correlative, however, we cannot rule out the alternative explanation: hemosporidian parasites might be more likely to infect individuals with elevated globulin levels, perhaps because they are weakened from a previous infection by other pathogens. Experimental infections would be necessary to substantiate the direction of this association.

This elevated globulin fraction appeared to be responsible for the increase in total plasma protein (and concomitant decrease in the Alb:Glo ratio) that we observed. Decreases in albumin (the most common protein in normal plasma) are generally associated with pathological states (Kilgas et al., 2006; Ots et al., 1998). However, albumin did not vary consistently with infection in this study: We observed a decrease only with respect to *Hemoproteus* burden. Moreover, we found scant evidence for a relationship between infection and other blood parameters (summarized in Table 1) that might have reflected immune response or stress (Davis, Maney, & Maerz, 2008). Results from other studies seeking links between these parameters and hemosporidian infections have also yielded inconsistent results. Some have documented positive correlations (e.g., in total WBC counts; Ots & Horak, 1998, lymphocytes; Ellis et al., 2014; Krams et al., 2013; Ots & Horak, 1998, and heterophils; Ellis et al., 2014; Krams et al., 2013), whereas others documented either no effect or a correlation in the opposite direction (e.g., in total WBC counts; Cornelius et al., 2014; Schoenle et al., 2017; Biard, Monceau, Motreuil, & Moreau, 2015, lymphocytes; Cornelius et al., 2014, heterophils, and H:L ratio; Cornelius et al., 2014; Ots & Horak, 1998). Considered in concert, these inconsistencies indicate that links between hemosporidian infections and total WBC cell count, heterophils, and lymphocytes are context dependent and not generalizable across host–parasite systems.

The host immune response is energetically costly, using resources that could have been allocated to other functions (Cornelius et al., 2014). An over‐reactive immune response can itself damage the host (Sorci, 2013). For example, increases in plasma protein (through the increase in globulins) could slow blood flow and reduce the oxygen transport of the blood, compounding the effects of erythrocyte destruction (Atkinson & Van Riper, 1991). However, we did not observe any negative effect of the host immune response (i.e., globulin levels) on our index of general health (body condition index) or short‐term survival. If mounting an immune response was costly, it was not detectable through these indices.

### 4.2 Pathogenicity of infection

Anemia (depletion of red blood cells) is a potential direct adverse effect of blood parasites on their hosts (LaPointe et al., 2012). Anemia can be measured in birds through hematocrit levels, where low hematocrit levels indicate a lower percentage of red blood cells in the total blood volume. This expectation of anemia is strongest among birds infected with *Plasmodium*, because merogony (asexual reproduction) occurs in the red blood cells and causes them to burst (Sorci, 2013). Abnormally low hematocrit has been linked to *Plasmodium* in many previous studies, involving both acute and chronic infections (Biard et al., 2015; Schoenle et al., 2017; Williams, 2005), although some studies involving chronic infections have not found a link (Marzal et al., 2008). Although merogony does not occur in the red blood cells of *Leucocytozoon* and *Hemoproteus*, these parasites still can cause anemia when they infect a high proportion of red blood cells with their gametocytes, making the red blood cells more fragile or more likely to be removed by the host immune system (Atkinson & Van Riper, 1991). In our study, however, only *Plasmodium* infection was associated with anemia: We found a strong, consistent relationship between *Plasmodium* prevalence and burden and low hematocrit levels. When infected with *Leucocytozoon* and *Hemoproteus*, birds did not exhibit lower hematocrit levels; indeed, the trend was in the opposite direction (Figure 3a), suggesting that these infections were not associated with anemia. These results were consistent with other studies that have failed to find a negative association between *Hemoproteus* or *Leucocytozoon* infection and hematocrit (reviewed in Fair, Whitaker, & Pearson, 2007), although these studies have generally focused on adult birds (Dawson & Bortolotti, 1997; Dufva, 1996; Marzal, de Lope, Navarro, & Moller, 2005; Ots & Horak, 1998).

Similarly, among the three genera, only *Plasmodium* was associated with lower short‐term survival: Infected birds were less likely to fledge than uninfected birds. The negative effect of *Plasmodium* on fledging success might have been mediated, at least in part, through low hematocrit levels, which can weaken a bird and make it more susceptible to other environmental stressors (LaPointe et al., 2012). For example, we found that birds with lower hematocrit levels were in poor condition (i.e., were lighter for a given body size) relative to birds with higher hematocrit (Figure 3b), and that birds that were in poor condition were less likely to fledge from the nest (Figure 3c). Low hematocrit levels and poor condition likely contributed to elevated nestling mortality in *Plasmodium*‐infected birds.

Infection with *Plasmodium* in the nestling stage also had long‐term survival consequences. Our mark–recapture analysis showed a negative association between nestling *Plasmodium* infections and postfledging survival probability within the first three years of life (Figure 4). This association, too, might have been mediated, at least in part, through the indirect effects of *Plasmodium* on body condition. Poor nestling body condition has previously been linked to disease mortality in crows, caused by a suite of bacterial, viral, and fungal infections, within three years after hatching (Townsend et al., 2010).

### 4.3 Mixed infections

Recent studies indicate that the consequences of infection can vary with the number of concurrent infections, both at the within‐species level and across broader taxonomic groups (Biard et al., 2015; Marzal et al., 2008). In this study, we compared the effects of multiple infections, defined here as infection by more than one genus of hemosporidian parasite, with the effects of single infections. Multiple infections and single infections yielded similar results, particularly for the plasma proteins (Table 2). Of note, globulins were highest among birds with multiple infections, lowest among uninfected birds, and intermediate among birds with single infections, indicating that the effect of these genera on antibody (Ig) production may be additive.

## 5 CONCLUSIONS

Our data suggest that acute infection by *Leucocytozoon*,* Plasmodium*, and *Hemoproteus* elicits an immune response in young crows, but that the pathogenicity varies across genera. *Plasmodium* was the only taxon with detectable costs, through its association with low hematocrit levels, poor body condition (indirectly), reduced fledging success, and reduced postfledging survival probability. When interpreting these results, however, it is important to note that analyses were conducted at the genus level and could obscure species‐ or lineage‐specific consequences. Within the *Leucocytozoon* alone, for example, we previously described 13 different lineages in this crow population (Freund et al., 2016), all of which were designated as a single taxon (*Leucocytozoon*) in the current study. Recent studies have indicated that pathogenicity can vary among hemosporidian lineages (Asghar et al., 2011; Ellis et al., 2014; Lachish et al., 2011), and that co‐infection by different lineages can have nonadditive effects (Marzal et al., 2008). Therefore, our categorization could have obscured species‐specific effects. Future analyses of lineage‐specific pathogenicity are warranted.

## CONFLICT OF INTEREST

None declared.

## AUTHORS’ CONTRIBUTIONS

AT and WB conceived the idea; AT wrote the manuscript; SW and AT collected data; SS, DF, and RS conducted parasite analyses. All authors contributed critically to the drafts and gave final approval for publication.

## DATA ARCHIVING

Data used in these analyses are available through the Dryad Digital Data Repository (https://doi.org/10.5061/dryad.8tt17dj).
