# Supplementary material for: Links between blood parasites, blood chemistry, and the survival of nestling American crows
Source: Ecol Evol. 2018 Aug 7;8(17):8779–90. doi: 10.1002/ece3.4287 (PMC6157653; doi:10.1002/ece3.4287)
Supplement: Supplementary file 1 [file ECE3-8-8779-s001.pdf]

Table S1. Descriptive statistics of hematological and biochemical measurements from American crow nestlings. Values are based on all samples collected, pooled across ages (0-40 days after hatching; mean age 17.69 ± 0.5 days). Estimates can include measurements collected from the same individual (minimum of three days between sampling periods).

Parameter	*n*	mean ± SE	range
WBC count	146	23438.9 ± 900.00	2500-60800
Heterophils	145	26.6 ± 1.32	2-75
Lymphocytes	147	34.54 ± 1.44	1-84
Hematocrit	148	29.8 ± 0.37	14-40
Plasma Protein	156	3.72 ± 0.06	7-2.2
Albumin	175	1.26 ± 0.03	0.3-2.3
Globulin	175	1.81 ± 0.05	0.6-5.2
