# Supplementary material for: Links between blood parasites, blood chemistry, and the survival of nestling American crows
Source: Ecol Evol. 2018 Aug 7;8(17):8779–90. doi: 10.1002/ece3.4287 (PMC6157653; doi:10.1002/ece3.4287)
Supplement: Supplementary file 2 [file ECE3-8-8779-s002.pdf]

Table S2. Full model output, including year, age, and sex (when significant or marginally significant), comparing the effects of infection prevalence (presence-absence of each genus) or co-infections (presence-absence of more than one genus; scored as no infections, single infections, or co-infections) on different blood parameters and fitness outcomes. Family group was included as a random effect in all models.

(1a) White blood cell count --- each genus

	Value	Std.Error	DF	t-value	p-value
(Intercept)	22852.773	2014.853	69	11.342152	0.0000
Leucocytozoon	-2383.998	2213.014	69	-1.077263	0.2851
Haemoproteus	4489.417	2392.813	69	1.876209	0.0649
Plasmodium	-140.878	2467.867	69	-0.057085	0.9546

(1b) White blood cell count --- co-infection

	Value	Std.Error	DF	t-value	p-value
(Intercept)	14914.439	4048.398	70	3.684035	0.0004
Co-infections	-1416.537	1443.795	70	-0.981121	0.3299
age	455.207	202.105	70	2.252333	0.0274

(2a) Heterophils --- each genus

	Value	Std.Error	DF	t-value	p-value
(Intercept)	40.02409	5.142335	68	7.783252	0.0000
Leucocytozoon	-2.08868	2.811074	68	-0.743020	0.4600
Haemoproteus	6.91406	3.281619	68	2.106905	0.0388
Plasmodium	-1.14600	3.285860	68	-0.348766	0.7283
age	-0.72682	0.258903	68	-2.807302	0.0065

(2b) Heterophils --- co-infection

	Value	Std.Error	DF	t-value	p-value
(Intercept)	38.00710	5.209608	70	7.295577	0.0000
Co-infections	0.11876	1.871927	70	0.063445	0.9496
age	-0.58882	0.259503	70	-2.269031	0.0264

(3a) Lymphocytes --- each genus

	Value	Std.Error	DF	t-value	p-value
(Intercept)	15.457426	6.106536	68	2.5312921	0.0137
Leucocytozoon	2.831879	3.401431	68	0.8325550	0.4080
Haemoproteus	-3.272678	3.982036	68	-0.8218605	0.4140
Plasmodium	1.870156	3.989568	68	0.4687615	0.6407
age	0.873628	0.309931	68	2.8187842	0.0063

(3b) Lymphocytes --- co-infection

	Value	Std.Error	DF	t-value	p-value
(Intercept)	16.881040	6.029015	70	2.7999668	0.0066
Co-infections	1.311870	2.211330	70	0.5932492	0.5549
age	0.776886	0.303383	70	2.5607391	0.0126

(4a) H:L --- each genus

	Value	Std.Error	DF	t-value	p-value
(Intercept)	0.3735509	0.16353736	68	2.2841929	0.0255
Leucocytozoon	-0.0703040	0.09013324	68	-0.7800004	0.4381
Haemoproteus	0.1681311	0.10535580	68	1.5958408	0.1152
Plasmodium	-0.0559062	0.10552115	68	-0.5298099	0.5980
age	-0.0256177	0.00826224	68	-3.1005773	0.0028

(4b) H:L --- co-infection

	Value	Std.Error	DF	t-value	p-value
(Intercept)	0.3177999	0.16422323	70	1.935170	0.0570
Co-infections	-0.0157220	0.05948716	70	-0.264293	0.7923
age	-0.0217850	0.00821206	70	-2.652805	0.0099

(5a) Hematocrit --- each genus

	Value	Std.Error	DF	t-value	p-value
(Intercept)	27.788751	1.4546698	72	19.103133	0.0000
Leucocytozoon	0.230427	0.8148279	72	0.282792	0.7781
Haemoproteus	-0.527059	0.9967433	72	-0.528781	0.5986
Plasmodium	-2.017636	0.9919904	72	-2.033927	0.0456
age	0.143550	0.0757040	72	1.896203	0.0619
sexM	-1.687557	0.7024231	72	-2.402479	0.0189

(5b) Hematocrit --- co-infection

	Value	Std.Error	DF	t-value	p-value
(Intercept)	27.677308	1.4566683	74	19.000419	0.0000
Co-infections	-0.495277	0.5580504	74	-0.887512	0.3777
age	0.146811	0.0752003	74	1.952274	0.0547
sexM	-1.561050	0.7000541 7	4	-2.229899	0.0288

(6a) Plasma protein --- each genus

	Value	Std.Error	DF	t-value	p-value
(Intercept)	3.365335	0.1027997	78	32.73681	0.0000
Leucocytozoon	0.267041	0.1098718	78	2.43048	0.0174
Haemoproteus	0.466828	0.1237266	78	3.77306	0.0003
Plasmodium	0.593684	0.1279099	78	4.64142	0.0000

(6b) Plasma protein --- co-infection

	Value	Std.Error	DF	t-value	p-value
(Intercept)	3.363324	0.10303212	80	32.64345	0
Co-infections	0.402275	0.06444057	80	6.24257	0

(7a) Albumin --- each genus

	Value	Std.Error	DF	t-value	p-value
(Intercept)	1.1287648	0.11479959	76	9.832481	0.0000
Leucocytozoon	0.0641282	0.06084213	76	1.054009	0.2952
Haemoproteus	0.0183241	0.06642703	76	0.275854	0.7834
Plasmodium	-0.0070075	0.06995435	76	-0.100173	0.9205
age	0.0126827	0.00557416	76	2.275263	0.0257
Year	-0.2566317	0.05800729	76	-4.424130	0.0000

(7b) Albumin --- co-infection

	Value	Std.Error	DF	t-value	p-value
(Intercept)	1.1299401	0.11345724	78	9.959171	0.0000
Co-infections	0.0340204	0.03801665	78	0.894880	0.3736
age	0.0128172	0.00549051	78	2.334424	0.0221
Year	-0.2602500	0.05382321	78	-4.835275	0.0000

(8a) Globulin --- each genus

	Value	Std.Error	DF	t-value	p-value
(Intercept)	1.2978363	0.1267555	77	10.238894	0.0000
Leucocytozoon	0.1661401	0.1036200	77	1.603360	0.1129
Haemoproteus	0.4341876	0.1107256	77	3.921296	0.0002
Plasmodium	0.4090041	0.1158843	77	3.529416	0.0007
Year	0.4179007	0.1082879	77	3.859165	0.0002

(8b) Globulin --- co-infection

	Value	Std.Error	DF	t-value	p-value
(Intercept)	1.3006308	0.12331876	79	10.546902	0e+00
Co-infections	0.3073582	0.06172547	79	4.979439	0e+00
Year	0.3872189	0.10337653	79	3.745714	3e-04

(9a) Alb:Glo --- each genus

	Value	Std.Error	DF	t-value	p-value
(Intercept)	1.1019357	0.05324421	77	20.695877	0.0000
Leucocytozoon	-0.0731872	0.05242089	77	-1.396147	0.1667
Haemoproteus	-0.1708518	0.05642781	77	-3.027794	0.0034
Plasmodium	-0.1206341	0.06202539	77	-1.944916	0.0554
Year	-0.3051324	0.05278843	77	-5.780290	0.0000

(9b) Alb:Glo --- co-infection

	Value	Std.Error	DF	t-value	p-value
(Intercept)	1.0943618	0.05204525	79	21.027120	0e+00
Co-infections	-0.1131747	0.02968658	79	-3.812320	3e-04
Year	-0.2895299	0.04943987	79	-5.856204	0e+00

(10) Body condition index --- each genus

	Value	Std.Error	DF	t-value	p-value
(Intercept)	4.521305	6.129801	126	0.737594	0.4621
Leucocytozoon	3.998852	5.033231	126	0.794490	0.4284
Haemoproteus	-3.841401	5.587774	126	-0.687465	0.4931
Plasmodium	1.105719	5.953223	126	0.185734	0.8530
Year	-22.212120	6.723315	126	-3.303745	0.0012

(11) Fledging success --- each genus

	Value	Std.Error	DF	t-value	p-value
(Intercept)	0.1954799	0.4910858	126	0.3980566	0.6913
Leucocytozoon	0.2538369	0.4049552	126	0.6268271	0.5319
Haemoproteus	-0.0785493	0.4704559	126	-0.1669643	0.8677
Plasmodium	-0.9515672	0.4580374	126	-2.0774878	0.0398
Year	1.1099154	0.5109416	126	2.1722941	0.0317
