# Supplementary material for: Links between blood parasites, blood chemistry, and the survival of nestling American crows
Source: Ecol Evol. 2018 Aug 7;8(17):8779–90. doi: 10.1002/ece3.4287 (PMC6157653; doi:10.1002/ece3.4287)
Supplement: Supplementary file 3 [file ECE3-8-8779-s003.pdf]

Table S3. Full model output, including year, age, and sex (when significant or marginally significant), comparing the effects of parasite burden (percentage of infected cells) on different blood parameters and fitness outcomes. Family group was included as a random effect in all models.

(1) White blood cell count

	Value	Std.Error	DF	t-value	p-value
(Intercept)	16166.631	4019.103	61	4.022448	0.0002
Leucocytozoon	-3878.126	2673.128	61	-1.450782	0.1520
Haemoproteus	-471.498	276.491	61	-1.705292	0.0932
Plasmodium	-354.112	370.094	61	-0.956817	0.3424
age	385.548	174.898	61	2.204414	0.0313

(2) Heterophils

	Value	Std.Error	DF	t-value	p-value
(Intercept)	0.4277604	0.17443081	60	2.452321	0.0171
Leucocytozoon	-2.66691	3.414382	60	-0.781081	0.4378
Haemoproteus	0.58393	0.354807	60	1.645769	0.1050
Plasmodium	0.77934	0.490537	60	1.588744	0.1174
age	-0.52835	0.213157	60	-2.478677	0.0160
Year	-7.60937	2.845310	60	-2.674357	0.0096

(3) Lymphocytes

	Value	Std.Error	DF	t-value	p-value
(Intercept)	16.077960	6.215012	61	2.586956	0.0121
Leucocytozoon	1.608458	3.932460	61	0.409021	0.6840
Haemoproteus	-0.356020	0.408480	61	-0.871572	0.3869
Plasmodium	-0.265071	0.527962	61	-0.502064	0.6174
age	0.904598	0.269326	61	3.358742	0.0014

(4) H:L

	Value	Std.Error	DF	t-value	p-value
(Intercept)	0.4277604	0.17443081	60	2.452321	0.0171
Leucocytozoon	-0.0492850	0.10546086	60	-0.467329	0.6420
Haemoproteus	0.0116698	0.01100247	60	1.060650	0.2931
Plasmodium	0.0163809	0.01453551	60	1.126955	0.2642
age	-0.0228881	0.00702064	60	-3.260113	0.0018
Year	-0.1980777	0.09187489	60	-2.155950	0.0351

(5) Hematocrit

	Value	Std.Error	DF	t-value	p-value
(Intercept)	30.120698	0.6803272	68	44.27384	0.0000
Leucocytozoon	0.916809	0.8320000	68	1.10193	0.2744
Haemoproteus	0.018621	0.0948122	68	0.19639	0.8449
Plasmodium	-0.512807	0.1152776	68	-4.44846	0.0000
sexM	-2.012021	0.6379653	68	-3.15381	0.0024

(6) Plasma protein

	Value	Std.Error	DF	t-value	p-value
(Intercept)	2.7695673	0.21879134	72	12.658487	0.0000
Leucocytozoon	-0.0146644	0.12119093	72	-0.121002	0.9040
Haemoproteus	0.0550630	0.01383210	72	3.980810	0.0002
Plasmodium	0.0427942	0.01663012	72	2.573294	0.0121
age	0.0425545	0.00954175	72	4.459821	0.0000

(7) Albumin

	Value	Std.Error	DF	t-value	p-value
(Intercept)	1.0909744	0.10600267	71	10.291952	0.0000
Leucocytozoon	-0.0057016	0.11066287	71	-0.051523	0.9591
Haemoproteus	0.0082128	0.00762886	71	1.076546	0.2853
Plasmodium	-0.0225608	0.00948953	71	-2.377445	0.0201
age	0.0154301	0.00460001	71	3.354350	0.0013
Year	-0.2341002	0.05776887	71	-4.052359	0.0001

(8) Globulin

	Value	Std.Error	DF	t-value	p-value
(Intercept)	1.5623670	0.10916533	72	14.311934	0.0000
Leucocytozoon	0.0450651	0.17149390	72	0.262779	0.7935
Haemoproteus	0.0488107	0.01231902	72	3.962224	0.0002
Plasmodium	0.0491393	0.01358879	72	3.616162	0.0006
Year	0.4278842	0.10555363	72	4.053714	0.0001

(9) Alb:Glo

	Value	Std.Error	DF	t-value	p-value
(Intercept)	0.9971921	0.04502754	72	22.146272	0.0000
Leucocytozoon	-0.0020604	0.10060837	72	-0.020479	0.9837
Haemoproteus	-0.0157678	0.00688214	72	-2.291120	0.0249
Plasmodium	-0.0207422	0.00852307	72	-2.433651	0.0174
Year	-0.3093577	0.05270271	72	-5.869864	0.0000

(10) Body condition index

	Value	Std.Error	DF	t-value	p-value
(Intercept)	6.822742	4.511738	123	1.512221	0.1330
Leucocytozoon	-7.564749	6.331183	123	-1.194840	0.2344
Haemoproteus	-0.161538	0.511522	123	-0.315799	0.7527
Plasmodium	-1.292727	0.786385	123	-1.643887	0.1028
Year	-31.551025	5.906110	123	-5.342099	0.0000

(11) Fledging success

	Value	Std.Error	DF	t-value	p-value
(Intercept)	-2.0896590	0.5245137	135	-3.983993	0.0001
Leucocytozoon	-0.2312309	0.5539655	135	-0.417410	0.6770
Haemoproteus	-0.0385439	0.0449748	135	-0.857011	0.3930
Plasmodium	0.0800639	0.0867197	135	0.923249	0.3575
age	0.1174508	0.0225767	135	5.202304	0.0000
